# A qualitative study of the stress autism mate app among autistic adults: user experiences and effects on stress awareness and coping skills

**DOI:** 10.1192/j.eurpsy.2026.11340

**Published:** 2026-07-31

**Authors:** E. Rotman, E. Keuning, on behalf of SAM-APP, B. Rekveld

**Affiliations:** GGz Centraal, Hilversum, Netherlands

## Abstract

**Introduction:**

mHealth applications offer promising solutions to structural challenges in mental health care—long waiting lists, high costs, and limited day-to-day support (Baños et al., 2022)—yet implementation is often difficult. This symposium presents the development, evaluation, and clinical use of the **Stress Autism Mate (SAM)** app and two spinoffs: **SAM Junior** and **Stappvoorstap**. SAM and SAM Junior are stand-alone stress-management apps co-created with clients of Emerhese Flevoland (GGz Centraal) using a Design Thinking approach (Van Asselt & Roke, 2025). The apps combine brief daily stress check-ins with autistic-moderated content to help users identify and reduce stress patterns. Effectiveness has been demonstrated across multiple studies, including a large randomized controlled trial (Hoeberichts et al., 2022, 2024; Spaargaren et al., 2025). SAM is now freely available in **34 countries** and **ten languages**. **Stappvoorstap**, developed with and for adults on mental-health waiting lists, adapts SAM to the waiting-time context. Ongoing mixed-methods research (e.g., Hoeberichts et al., 2025; Van Asselt et al., 2025b) informs continuous (re)design and scaling across countries, cultures, and populations. The symposium will review key iterative design decisions and provide a balanced synthesis of findings, offering practical guidance for effective development and integration of mHealth interventions within and alongside routine mental-health care.

**Objectives:**

This study explored how autistic outpatient adolescents and adults experience the use of SAM in relation to stress awareness, coping behaviours, and engagement with digital tools.

**Methods:**

A qualitative phenomenological design was used, involving in-depth interviews with ten autistic participants (N = 10) who used SAM for at least four weeks. Reflexive thematic analysis was conducted to identify key patterns in user experiences.

**Results:**

Three key processes were identified: (1) SAM facilitated increased awareness of previously unrecognised stress by externalising internal states, (2) participants shifted from avoidant to active coping strategies, supported by structured reflection and coping suggestions, and (3) the app’s emotionally neutral, predictable design created a safe and engaging space for self-regulation. However, tensions between structure and flexibility highlighted the need for greater personalisation to sustain engagement over time.

**Conclusions:**

In conclusion, SAM supports autistic individuals in transforming vague stress experiences into actionable insights, fostering emotional literacy and coping capacity. These findings extend prior quantitative evidence on SAM’s efficacy and provide actionable design recommendations for mHealth interventions aimed at neurodivergent populations.

**Disclosure of Interest:**

None Declared

